# PATHOS: a phase II/III trial of risk-stratified, reduced intensity adjuvant treatment in patients undergoing transoral surgery for Human papillomavirus (HPV) positive oropharyngeal cancer

**DOI:** 10.1186/s12885-015-1598-x

**Published:** 2015-08-27

**Authors:** Waheeda Owadally, Chris Hurt, Hayley Timmins, Emma Parsons, Sarah Townsend, Joanne Patterson, Katherine Hutcheson, Ned Powell, Matthew Beasley, Nachi Palaniappan, Max Robinson, Terence M. Jones, Mererid Evans

**Affiliations:** 1 Velindre NHS Trust, Velindre Road, Cardiff, CF14 2TL UK; 2 Wales Cancer Trials Unit, 6th Floor, Neuadd Meirionnydd, Cardiff University, Heath Park, Cardiff, CF14 4YS UK; 3 Mount Vernon Hospital, Rickmansworth Road, Northwood, HA6 2RN UK; 4 Research and Development Office, 3rd Floor, Velindre NHS Trust, 14 Cathedral Road, Cardiff, CF11 9LJ UK; 5 Institute of Health and Society, Newcastle University, The Baddiley-Clark Building, Richardson Road, Newcastle-upon-Tyne, NE2 4AX UK; 6 Speech and Language Therapy, Department of Head and Neck Surgery, Section of Speech Pathology and Audiology, MD Anderson Cancer Centre, Houston, Texas USA; 7 HPV Research Group, Section of Pathology, Cardiff University School of Medicine, UHW Main Building, Heath Park, Cardiff, CF14 4XN UK; 8 Bristol Cancer Institute, University Hospitals Bristol NHS Foundation Trust, Horfield Road, Bristol, BS2 8ED UK; 9 Centre for Oral Health Research, Newcastle University, Framlington Place, Newcastle-upon-Tyne, NE2 4BW UK; 10 Head and Neck Surgery, Department of Molecular and Clinical Cancer Medicine, Institute of Translational Medicine, University of Liverpool, 200 London Road, Liverpool, L3 9TA UK

## Abstract

**Background:**

Human papillomavirus-positive oropharyngeal squamous cell carcinoma is increasing in incidence worldwide. Current treatments are associated with high survival rates but often result in significant long-term toxicities. In particular, long-term dysphagia has a negative impact on patient quality of life and health. The aim of PATHOS is to determine whether reducing the intensity of adjuvant treatment after minimally invasive transoral surgery in this favourable prognosis disease will result in better long-term swallowing function whilst maintaining excellent disease-specific survival outcomes.

**Methods/Design:**

The study is a multicentre phase II/III randomised controlled trial for patients with biopsy-proven Human papillomavirus-positive oropharyngeal squamous cell cancer staged T1-T3 N0-N2b with a primary tumour that is resectable via a transoral approach. Following transoral surgery and neck dissection, patients are allocated into three groups based on pathological risk factors for recurrence. Patients in the low-risk pathology group will receive no adjuvant treatment, as in standard practice. Patients in the intermediate-risk pathology group will be randomised to receive either standard dose post-operative radiotherapy (control) or reduced dose radiotherapy. Patients in the high-risk pathology group will be randomised to receive either post-operative chemoradiotherapy (control) or radiotherapy alone. The primary outcome of the phase II study is patient reported swallowing function measured using the MD Anderson Dysphagia Inventory score at 12 months post-treatment. If the phase II study is successful, PATHOS will proceed to a phase III non-inferiority trial with overall survival as the primary endpoint.

**Discussion:**

PATHOS is a prospective, randomised trial for Human papillomavirus-positive oropharyngeal cancer, which represents a different disease entity compared with other head and neck cancers. The trial aims to demonstrate that long-term dysphagia can be lessened by reducing the intensity of adjuvant treatment without having a negative impact on clinical outcome. The study will standardise transoral surgery and post-operative intensity-modulated radiotherapy protocols in the UK and develop a gold-standard swallowing assessment panel. An associated planned translational research programme, underpinned by tumour specimens and sequential blood collected as part of PATHOS, will facilitate further empirical understanding of this new disease and its response to treatment.

**Trial registration:**

This study is registered with ClinicalTrials.gov identifier NCT02215265.

## Background

Oropharyngeal squamous cell carcinoma (OPSCC) is a rapidly increasing disease in the UK and other developed countries as a result of Human papillomavirus (HPV) genotype 16 infection. Currently, over 70 % of OPSCC in Europe is HPV 16 positive [1]. HPV status is a strong and independent prognostic factor for survival, and HPV-positive OPSCC has a 58 % reduction in the risk of death compared to HPV-negative OPSCC [2]. Other factors known to influence prognosis in HPV-positive OPSCC include smoking, particularly current smoking, nodal stage and patient comorbidities [2–4].

Currently, the management of OPSCC is based on the stage of disease as well as clinician and patient preference, irrespective of HPV status. Early stage disease is treated with either surgery or radiotherapy (RT) alone, whilst locally advanced disease requires multimodality treatment with primary chemoradiotherapy (CRT) +/- neck dissection or primary surgical resection followed by post-operative RT/CRT. Severe late toxicities after multimodality treatment are reported in up to 43 % of patients and may be permanent [5]. Patients and their carers report dysphagia to be a primary cause for distress, and patient reported dysphagia independently predicts for poor long-term quality of life (QOL) [6, 7]. Patients with HPV-positive OPSCC tend to be young (mean age 54 years) and fit at presentation [2]. Reducing the adverse impact of treatment on function and maintaining good QOL are therefore of paramount importance in these patients who have good prognosis disease.

### Role of transoral surgery

With the advent of minimally invasive techniques, such as Transoral Laser Microsurgery (TLM) and Transoral Robotic Surgery (TORS), there has been renewed interest in primary surgical treatment for OPSCC as these techniques result in less morbidity when compared to open surgery. There are as yet no prospective randomised data on TLM/TORS for OPSCC but retrospective studies have demonstrated excellent outcomes. A US study of 204 patients with stage III-IV OPSCC treated with TLM and neck dissection found rates of local control (LC), overall survival (OS) and disease-free survival (DFS) to be 97, 86 and 82 % respectively at 3 years, with HPV-positive OPSCC having even better outcomes [8]. Most patients had adjuvant treatment (RT/CRT), which increased toxicity. Single centre data on TLM in the UK is also encouraging. Data from Liverpool on 153 patients with T1-T3 OPSCC (66 % HPV positive) treated with TLM and neck dissection demonstrate 3 year OS of 84.5 %, disease specific survival (DSS) of 91.7 % and DFS of 78.2 %. Patients with HPV-positive OPSCC had a 71 % reduction in the risk of death. 83.6 % of patients received adjuvant treatment. (TMJ, manuscript submitted). Single institution data also show a functional advantage with upfront transoral surgery compared to primary CRT [9]. A UK study compared swallowing function between 23 patients with locally advanced OPSCC treated with TLM +/- adjuvant therapy and 33 matched patients treated with CRT from a historical cohort and reported improved early swallowing function at 3 months in the upfront surgery group, using 3 different swallowing measures, including the MD Anderson Dysphagia Inventory (MDADI) score [10].

### Adjuvant therapy: risk factors, RT dose and use of chemotherapy

Currently, decisions about adjuvant therapy after surgery are based on the presence of pathological risk factors established more than 20 years ago in studies that included squamous cancers from multiple head and neck anatomical subsites and that did not test for tumour HPV status [11]. These risk factors include surgical margin status, presence of perineural and vascular invasion, number of lymph node metastases and presence of extracapsular spread (ECS) of nodal disease. The relevance of these risk factors in HPV-positive disease has been questioned and the optimum adjuvant treatment protocols for HPV-positive OPSCC are yet to be determined [12].

Adjuvant RT after surgery for advanced head and neck cancers improved LC in the RTOG 73-03 trial [13]. Subsequent studies recommended a minimum dose of 57.6Gy to the primary site and involved nodal areas and doses of up to 63Gy to areas of ECS [14]. However, lower doses of adjuvant RT may be sufficient for HPV-positive OPSCC based on the following: (i) observations that HPV-positive cell lines show increased radiosensitivity compared to HPV-negative cell lines in vitro [15, 16]; (ii) phase II data (ECOG 1308) showing equivalent LC rates at 2 years with reduced dose RT (54Gy in 27 × 2Gy fractions) in patients with HPV-positive OPSCC who achieved a complete response after 3 cycles of induction chemotherapy [17]; (iii) use of lower doses of prophylactic RT (50Gy in 35 × 1.4Gy fractions, equivalent to 43Gy in 2Gy fractions) with no increase in recurrences [18].

The EORTC 22931 and RTOG 9501 Randomised Controlled Trials (RCTs) showed that adjuvant CRT improved LC and DFS compared with adjuvant RT alone in some patients with advanced head and neck cancer [19, 20]. When the results of both studies were pooled, adjuvant CRT significantly improved OS in patients with positive (or ‘involved’) surgical margins and/or presence of nodal ECS [21]. These pathological features are now widely used criteria for adjuvant CRT. There is variation in practice however, particularly around the issue of surgical margins with positive (<1 mm) and close (1-5 mm) margins being grouped together in some studies. A survey of clinical oncologists from 17 UK centres reported that all would recommend adjuvant CRT for positive surgical margins (<1 mm) whilst only 30 % would advocate its use for close margins (1–5 mm) and 88 % for ECS [22]. The relevance of the EORTC 22931 and RTOG 9501 study results should be questioned in the context of HPV-positive OPSCC. Patients in these studies had tumours from multiple head and neck anatomical subsites and the prevalence rate of HPV-positive OPSCC would have been significantly lower than the current rate [1]. Evidence for using adjuvant CRT after transoral surgery for OPSCC is also lacking. A retrospective study of TLM for advanced OPSCC showed that the addition of chemotherapy to adjuvant RT did not improve outcomes even in the presence of ECS, possibly because a high proportion of patients in that study had HPV-positive tumours with already excellent outcomes [8, 12].

### Dysphagia after treatment

There are several factors that may contribute to dysphagia after treatment. Dysphagia after RT has been shown to correlate with increased mean doses of radiation to swallowing-related organs, with a higher mean dose to superior pharyngeal constrictor muscle region and larynx being particularly associated with worse long-term swallowing outcomes [23, 24]. The sigmoidal shape of the normal tissue complication probability curve indicates that increasing mean dose to pharyngeal musculature between 50Gy and 60Gy is a critical point at which risk of long-term dysphagia begins to inflect upwards, suggesting that reducing radiation dose from 60Gy to 50Gy could impart a clinically significant improvement in long-term swallowing outcomes [23]. Adding concurrent chemotherapy to primary or adjuvant RT increases the risk of dysphagia: a systematic review of TORS for OPSCC showed clear demarcation in swallowing outcomes across a variety of outcome measures in patients who received adjuvant RT alone compared to adjuvant CRT [25].

#### Study rationale

Current treatments for HPV-positive OPSCC are associated with high survival rates but often result in significant long-term toxicities, particularly affecting swallowing function, that have a negative effect on QOL. Patients recruited into PATHOS will undergo transoral surgery to resect their primary tumours as well as a neck dissection. Post-operatively, they will be stratified into risk groups according to the presence or absence of pathological risk factors for recurrence. The aim of PATHOS is to determine whether reducing the intensity of adjuvant treatment after minimally invasive surgery in HPV-positive OPSCC, either by lowering RT dose in intermediate-risk patients or omitting chemotherapy in high-risk patients will result in better swallowing function, whilst maintaining excellent clinical outcomes. The primary outcome of the PATHOS phase II study will be long-term patient reported swallowing function measured using the MDADI score at 12 months post-treatment. Secondary outcomes will include local control rates and survival as well as a panel of objective and self-reported swallowing assessments. If the phase II study is successful, PATHOS will continue to a phase III study. The primary outcome of the proposed phase III study will be overall survival.

## Methods/Design

### Study design

**Fig. 1:**
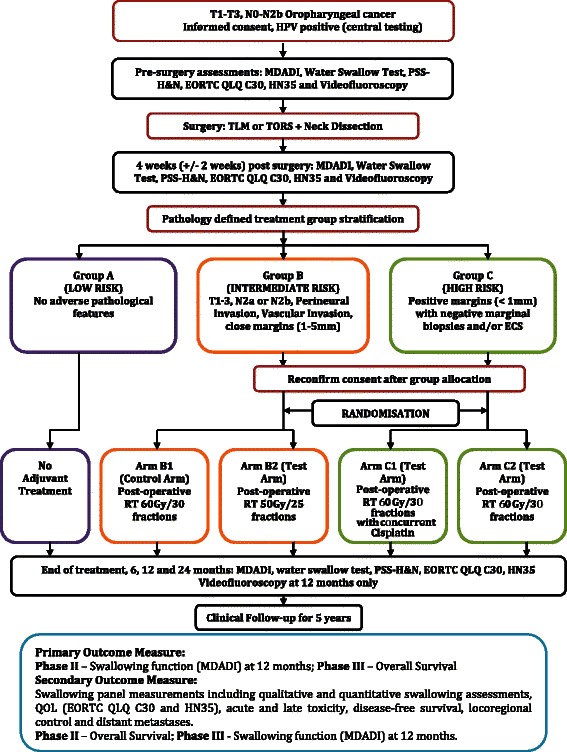
Trial schema

PATHOS is a multicentre, open label, parallel group phase II/III RCT funded by Cancer Research UK (CRUK). Patients must have biopsy proven OPSCC and should be clinically staged T1–T3 N0-N2b M0. Their primary tumour must be considered resectable via a transoral approach as determined by the local Multidisciplinary Team (MDT). HPV-positivity will be confirmed by central testing of diagnostic biopsy specimens by p16 immunohistochemistry and high risk HPV in-situ hybridisation. Synchronous neck dissection will be undertaken as per standard protocols depending upon the volume of regional metastatic disease. Following surgery, patients will be allocated into study groups based on histological findings (Fig. 1):

Group A: Patients whose tumours have no adverse histological features will not receive any adjuvant treatment as per standard of care.

Group B: Patients with T3 tumours (or T1–T2 tumours with additional risk factors), N2a (metastasis in single ipsilateral node 31–60 mm diameter) or N2b (metastasis in multiple ipsilateral nodes <61 mm diameter), tumours with evidence of perineural and/or vascular invasion, or close margins (1–5 mm) around the primary tumour specimen but with negative marginal biopsies and no evidence of cervical lymph node ECS. Patients in this group will be randomised to either post-operative RT 60Gy in 30 fractions over 6 weeks (Control Arm B1) or post-operative RT 50Gy in 25 fractions over 5 weeks (Test Arm B2).

Group C: Patients with tumours of any T or any N stage with the following high risk pathological features: positive (<1 mm) margins around the primary tumour (but with negative marginal biopsies) and/or evidence of cervical lymph node ECS. Patients in this group will be randomised to either post-operative CRT 60Gy in 30 fractions over 6 weeks with concurrent cisplatin (Control Arm C1) or post-operative RT 60Gy in 30 fractions over 6 weeks without chemotherapy (Test Arm C2).

### Participating sites

The phase II trial will recruit at over approximately 20 sites in the UK. Participating sites will be required to complete a registration form to confirm that they have adequate resources and experience to conduct the trial. The planned phase III trial will recruit across Europe.

### Participant eligibility

Participants are eligible to enter the trial pre-operatively if they meet all of the inclusion criteria and none of the exclusion criteria (Table 1). Post-operatively, patients allocated to Groups B and C on the basis of their pathology must re-confirm their consent for the study and will be assessed for their suitability for adjuvant treatment. Patients in Group B must be fit to undergo RT. Patients in Group C must be fit to undergo CRT and meet additional criteria as per Table 2.

**Table 1:** Inclusion and exclusion criteria for all patients in PATHOS trial

Inclusion criteria for all patients	
1.	Histologically confirmed diagnosis of OPSCC
2.	HPV-positive on central testing
3.	Stage T1-T3, N0-N2b tumours (based on cross-sectional imaging investigations carried out within 6 weeks of study entry)
4.	Local MDT decision to treat with primary transoral resection and neck dissection
5.	Fit for surgery and adjuvant treatment as assessed by the local MDT
6.	Aged 18 or over
7.	Able to provide written informed consent
Exclusion criteria for all patients	
1.	HPV-negative tumours
2.	Stage T4 tumours and/or T1–T3 tumours where transoral surgery is considered not feasible
3.	N2c–N3 nodal disease
4.	Unresectable retropharyngeal node involvement
5.	Current smokers with N2b disease including smokers up to 2 years before diagnosis
6.	Any pre-existing medical condition likely to impair swallowing function and/or a history of pre-existing swallowing dysfunction prior to index oropharyngeal cancer
7.	Patients with distant metastatic disease (stage IVc)
8.	Patients with a history of malignancy in the last 5 years, except basal cell carcinoma of the skin or carcinoma in-situ of the cervix
9.	Women who are pregnant or breastfeeding and fertile women who will not be using contraception during the trial

**Table 2:** Additional inclusion and exclusion criteria for patients in Group C

Inclusion criteria for patients in Group C	
1.	Bone marrow reserve adequate for chemotherapy (i.e. absolute neutrophil count (ANC) ≥ 1.5 × 10^9^/l and platelet count ≥ 100 × 10^9^/l)
2.	Adequate creatinine clearance defined as GFR ≥ 50 ml/min
Exclusion criteria for patients in Group C	
1.	History of significant cardiac or other medical conditions that preclude the use of cisplatin and intravenous hydration
2.	Clinically significant hearing impairment sufficient to affect daily living and/or pre-existing tinnitus
3.	Pre-existing peripheral neuropathy that precludes the use of cisplatin
4.	Hypersensitivity to the active substance or other platinum compounds or to any of the other excipients
5.	Dehydrated condition

### Method of randomisation

Patients in Groups B and C will be randomised to a trial arm using the method of minimisation with a random element. Randomisation will be performed centrally by the Wales Cancer Trials Unit (WCTU). Participants will be stratified prior to randomisation by T stage, N stage, smoking history and treating centre.

### Study Interventions

#### Surgery

Surgery to the primary site will be carried out by TLM or TORS, while a standard open approach will be used for neck dissection. These can be carried out as a single or staged procedure. Surgery should take place within 4 weeks (maximum 6 weeks) of study registration.

##### Transoral Laser Microsurgery (TLM) and marginal biopsies

TLM will be conducted according to the principles outlined by Steiner and Ambrosch [26]. Tumours will be removed in several (at least two) planned pieces following trans-tumoural resection. It is mandatory that representative marginal biopsies are taken from the tumour bed in all cases of TLM to ensure complete surgical removal of the tumour. Re-resection is allowed when initial marginal biopsies are found to be positive for microscopic disease. If positive marginal biopsies are obtained on re-resection, the patient is withdrawn from the trial.

##### Transoral Robotic Surgery (TORS)

TORS involves *en bloc* removal of the tumour as per the principles outlined in the da Vinci Transoral Surgery Procedure Guide:

http://www.uphs.upenn.edu/pennorl/education/documents/daVinciTORSProcedureGuide.pdf. As tumours are typically removed *en bloc*, marginal biopsies are usually not required.

##### Neck Dissection

Patients with clinically node negative (cN0) disease will undergo a selective neck dissection involving clearance of at least nodal levels II and III. Patients with clinically node positive (cN+) disease will undergo clearance of lymph node levels II and III and any additional involved lymph node levels. Patients with neck disease involving adjacent structures will undergo a modified radical neck dissection. In the case of non-lateralised primary tumours, as an alternative to non-surgical treatment (see below), some centres may undertake a selective neck dissection of the contralateral cN0 neck.

#### Radiotherapy

Patients should start RT within 5 weeks and no later than 6 weeks from surgery, so that combined treatment (surgery and RT) is completed within 11 weeks to avoid poor LC and survival rates that result from protracted treatment [11]. Patients are managed as category 1 as per the Royal College of Radiologists Guidelines and RT should be completed within 6 weeks for patients having 60Gy in 30 fractions and within 5 weeks for those having 50Gy in 25 fractions [27].

The primary tumour should be categorised as lateralised or non-lateralised based on clinical and radiological assessments.

- Lateralised tumour: Tonsillar tumour confined to the tonsillar fossa or extending onto or into the adjacent base of tongue and/or soft palate by less than 1 cm.
- Non-lateralised tumour: Tonsillar tumour that involves the adjacent base of tongue and/or soft palate by more than 1 cm or a tumour that arises from a midline structure (base of tongue, soft palate, posterior pharyngeal wall).

Patients with lateralised tumours should undergo unilateral neck RT, regardless of the nodal stage of the ipsilateral neck. Patients with non-lateralised tumours should undergo bilateral neck RT, except in cases where they have undergone contralateral selective neck dissection and pN0 status is confirmed on that side (see above).

PATHOS uses a geometric approach to define target volumes. Pre-operative imaging, pan-endoscopy reports, operative findings and pathology information should be used to delineate target volumes. The Clinical Target Volume 1 (CTV1) includes the primary and nodal tumour beds with a margin (1–1.5 cm) and all pathologically involved nodal levels. Arms B1, C1, C2 receive 60Gy/30 fractions and Arm B2 receives 50Gy/25 fractions. The Clinical Target Volume 2 (CTV2) includes all at risk uninvolved nodal levels that require prophylactic RT. Arms B1, C1, C2 receive 54Gy/30 fractions and Arm B2 receives 50Gy/25fractions. Some centres may, *a priori* opt to boost high-risk sub-volume(s) for patients in Group C to 66Gy/30 fractions. Neck node levels for prophylactic RT should be outlined according to updated consensus guidelines and atlas [28]. A margin (3–5 mm) will be added to each CTV to produce the respective Planning Target Volume.

Dose constraints to the following organs at risk will be used for treatment plan optimisation: spinal cord, brainstem, parotid glands. Investigators are also encouraged to contour swallowing-related structures. These include the pharyngeal constrictor muscles (superior, middle and inferior), supraglottic/glottic larynx, cricopharyngeus, oesophageal inlet, cervical oesophagus and oral cavity and should be outlined according to the PATHOS atlas of swallowing structures, itself based on previously published guidelines [24, 29]. The swallowing structures will not be used for treatment plan optimisation but swallowing outcomes will be correlated to the dose received by these structures. All patients will be planned using Intensity Modulated Radiotherapy (IMRT).

#### Chemotherapy

The following regimens can be used: Cisplatin 100 mg/m^2^ administered intravenously in a three weekly-cycle on days 1 and 22 of the RT schedule or Cisplatin 40 mg/m^2^ weekly for a maximum of 6 weeks. Carboplatin may be used instead of Cisplatin from cycle 2 onwards if the patient develops complications (ototoxicity, impaired renal function) related to Cisplatin.

### Assessments and outcomes

Comprehensive assessment of swallowing function requires a multidimensional panel of measures that incorporates instrumental examination of swallowing along with clinician-rated and patient reported outcomes. A functional outcomes panel for assessing swallowing function has been developed for PATHOS (Table 3). The following assessments will be conducted prior to surgery, 4 weeks post surgery and at 4 weeks, 6, 12 and 24 months post treatment: (1) MD Anderson Dysphagia Inventory score (MDADI); (2) Water swallow test (WST); (3) Performance Status Scale-Head and Neck (PSS-HN); (4) Quality of Life questionnaires (EORTC QLQ-C30 and EORTC QLQ-H&N35). Patients will also undergo a videofluoroscopy (VF) assessment prior to surgery, at 4 weeks post surgery and at 12 months post treatment. CTCAE Toxicity (v4.03) will be assessed weekly during and at the end of RT and at 4 weeks, 6, 12 and 24 months.

**Table 3:** Functional outcomes panel for multidimensional assessment of swallowing function

Study	Description	Domain	Endpoint
MDADI	MDADI is a patient reported swallowing outcome measure, specifically designed and psychometrically validated for the head and neck cancer population	Swallowing-related QOL	Total/Composite, Global, Subscale Scores (continuous scores: 20 to 100)
WST	100mls WST is a timed swallowing test.	Swallow performance	Swallow capacity (mls per swallow)
			Swallow volume (mls per swallow)
VF	VF is the gold standard radiographic measure of swallowing function. It allows quantification of more objective endpoints of swallowing function including pathophysiology, swallowing efficiency and airway protection.	Swallow physiology	MBSImpairment profile (MBSImp) (continuous scores: oral impairment 0 to 22; pharyngeal impairment 0 to 29) [33]
		Airway protection	Penetration-aspiration scale (PAS) (ordinal score: 1 to 8)
			Aspiration, yes/no (binary) [34]
		Pharyngeal dysphagia grade	Videofluoroscopic Swallow Grade-Head & Neck (VSG-HN) (ordinal grade: 0 to 4)
PSS-HN	PSS-HN is a 3-item scale designed to evaluate functional performance of head and neck cancer patients according to normalcy of diet, eating in public and understandability of speech	Functional performance status	Normalcy of diet subscale, public eating subscale, understandability of speech scores (ordinal: 0 to 100)
EORTC QLQ C30 H&N35	QOL questionnaires	Health related QOL	Raw scores from scales and single item measures are transformed to a standardised 0–100 final scale score.

#### Primary outcome measures

The primary outcome of the phase II study will be swallowing function, measured using the 19-item composite MDADI score at 12 months post-treatment. The primary outcome of the planned phase III study will be overall survival (OS).

#### Secondary outcome measures

These will include data from: (1) functional outcomes panel (Table 3); (2) acute and late toxicity using CTCAE version 4.03; (3) QOL using EORTC QLQ C30 and H35 questionnaires; (4) overall survival (OS); (5) disease free survival (DFS); (6) locoregional control (LC); (7) distant metastases

### Sample size calculation

#### Phase II

Data show that a 10-point difference in mean MDADI score can differentiate aspirators from non-aspirators, tube-dependent from oral eaters and clinically distinct diet levels [30]. For the study to have 80 % power to detect this difference (two sided 5 % alpha), a sample size of 148 patients is required (i.e*n* = 74 in both randomisations). Given a 20 % loss to follow up as shown in previous studies, 186 patients will need to be randomised [20]. Assuming that 15 % of patients recruited are not randomised post-operatively (10 % who do not require adjuvant treatment and 5 % who decline randomisation) and that 10 % who are consented to the study will not be HPV positive at central HPV testing, we estimate that 242 patients will need to be enrolled into the phase II study.

#### Phase III

If the trial proceeds to phase III, then a sample size calculation for non-inferiority will be made. It is likely that around 800 patients will need to be recruited to prove that survival is maintained with de-intensified adjuvant therapy and European collaboration (through the EORTC) will be required for this.

### Statistical analyses

Mean MDADI scores at 12 months will be compared between arms using either a t-test or nonparametric methods depending upon distributions. We will adjust for the randomisation stratification variables using regression techniques. This primary analysis will be conducted when the last patient has had their 12 month assessment. An Independent Data Monitoring Committee will review the accumulating data (survival, toxicities, recruitment) at 6 monthly intervals. Strict monitoring has been built in for recurrence. A formal interim analysis will be performed after 38 patients have been randomised in each randomisation (19 per arm), treated and followed up for 6 months. Within each randomisation, a stopping rule will be based on observing an absolute observed difference of 6 or more locoregional recurrences and/or deaths in either of the intervention arms. For swallowing endpoints, subgroup analysis by T stage and tumour subsite (tonsil, soft palate, tongue base) will be carried out, as the most likely relevant clinical co-variables affecting swallowing function*.*

### Quality Assurance (QA)

All surgeons will need to demonstrate evidence of suitable training in the procedures employed and/or an established surgical practice in the relevant techniques (TLM/TORS). They should have undertaken a minimum of 5 previous transoral resections for OPSCC. It is expected that over the duration of the trial, positive marginal biopsy rates for an individual surgeon will not exceed 10 %.

The Radiotherapy Quality Assurance (RTQA) programme for the trial will be coordinated by the National Radiotherapy Trials Quality Assurance group. A comprehensive RTQA guidance document has been developed to accompany the main trial protocol. In brief, this will consist of pre-accrual and on-trial components. Each site must perform a pre-accrual outlining benchmark case on one lateralised and one non-lateralised case. Sites may need to complete a pre-accrual planning exercise of a benchmark case, depending on participation in other national head and neck trials. Real time review of the first lateralised and non-lateralised patients recruited by each centre will be carried out before treatment starts, both for outlining and planning.

All swallow assessments will be conducted by speech and language therapists with the required level of competency, or appropriately trained research nurses. DVDs of the VFs will be assessed centrally by members of the research team to QA the functional endpoint data.

### Translational research

The trial is associated with a CR-UK funded bioresource collection – PATHOS-T. Accordingly, up to five geographically distinct biopsies from the primary tumour will be harvested prior to surgical resection. In addition, up to two samples of involved cervical lymph node tissue will also be collected. Blood samples for research will also be taken before treatment and at 6 weeks, 6, 12, 18 and 24 months post treatment. Trial participants will be asked for additional optional consent to participate in this aspect of the study.

### Regulatory approval, sponsorship and current status

PATHOS has ethical approval from the Wales Research Ethics Committee which is legally recognised by the UK Ethics Committee Authority for review and approval. It also has approval from the Medicines and Health Care Product Regulatory Agency to be conducted in the UK. The Wales Cancer Trials Unit, a CRUK core funded and UK Clinical Research Collaboration accredited Clinical Trials Unit, is coordinating the trial. Velindre NHS Trust is the sponsor for the trial. A Trial Steering Committee and an Independent Data Monitoring Committee has been set up to monitor the progress and safety of the study. The PATHOS Trial Management Group, including clinicians, clinical trial unit staff, patient representatives, nursing and pharmacy representatives carry out the day-to-day running of the trial. PATHOS is registered with ClinicalTrials.gov identifier: NCT02215265.

## Discussion

A systematic review and meta-analysis of more than 500 OPSCC patients treated with TORS in 17 retrospective studies concluded that minimally invasive surgical techniques had a positive effect on QOL and long-term function as well as good oncological control [31]. The authors suggested that there was potential to reduce the intensity of treatment based on successful surgical control of disease in good prognosis HPV-positive patients. However, further validation through RCTs, like PATHOS, is needed prior to widespread shifts in practice. The RT dose of 50Gy in 25 fractions in the test arm (B2) of PATHOS was recommended by the National Cancer Institute Head and Neck Cancer Steering Committee Clinical Trials Planning Meeting on transoral resection of pharyngeal cancer [32]. This reduced dose is also currently being used in a parallel US study (ECOG 3311) for transorally resected HPV-positive OPSCC. Another ongoing US study (ADEPT) is investigating if concurrent chemotherapy can be withheld in patients with ECS in the adjuvant setting. PATHOS is the only study to investigate both the effects of lowering RT dose and omitting chemotherapy in the same study.

PATHOS will allow clinical and pathological correlations of outcomes for HPV-positive disease, such that predictive factors for disease behaviour can be determined specifically in the context of HPV-positive disease. The study also provides a unique opportunity in the UK to standardise transoral surgical approaches for the treatment of OPSCC. It is imperative that in light of the increased uptake of these new techniques that surgical QA be established. This will ensure the rigorous application of appropriate and consistent surgical standards to allow valid comparison whenever these techniques are used in surgical trials and more importantly whenever they are used to treat patients. A panel of objective and self-reported swallowing assessments has been developed for PATHOS to allow multidimensional assessment of swallowing function. This panel will be prospectively validated in the trial and represents a step change in the standardisation of swallowing assessment in head and neck trials. Equally important is the fact that PATHOS will be the first UK study of post-operative IMRT for head and neck cancer. A novel aspect of the study will be outlining of the swallowing structures by investigators in participating centres. Dose/volume data for swallowing structures will be correlated with long-term swallowing function, collected prospectively in this multicentre randomised trial.

PATHOS phase II will open to recruitment in the UK in June 2015, with a planned recruitment period of 3 years. If the phase II study is successful, we plan to proceed to a phase III study to establish survival non-inferiority in the de-intensified treatment arms, which will require European collaboration.

## Abbreviations

PATHOS: Post-operative adjuvant treatment for HPV-positive tumours
CRT: Chemoradiotherapy
CRUK: Cancer Research UK
CTCAE: Common terminology criteria for adverse events
CTV: Clinical target volume
DFS: Disease-free survival
DSS: Disease-specific survival
ECS: Extracapsular spread
HPV: Human papillomavirus
IMRT: Intensity modulated radiotherapy
LC: Local Control
MDADI: MD Anderson dysphagia inventory
MDT: Multidisciplinary Team
OPSCC: Oropharyngeal squamous cell carcinoma
OS: Overall survival
PSS-HN: Performance status scale – head and neck
QOL: Quality of life
QA: Quality assurance
RCT: Randomised controlled trial
RT: Radiotherapy
TLM: Transoral laser microsurgery
TORS: Transoral robotic surgery
VF: Videofluoroscopy
WCTU: Wales Cancer trials unit
WST: Water swallow test

## References

[1] Mehanna H, Beech T, Nicholson T, El-Hariry I, McConkey C, Paleri V (2013). Prevalence of Human papillomavirus in oropharyngeal and non-oropharyngeal head and neck cancer – systematic review and meta-analysis of trends by time and region. Head Neck.

[2] Ang KK, Harris J, Wheeler R, Weber R, Rosenthal DI, Nguyen-Tan PF (2010). Human papillomavirus and survival of patients with oropharyngeal cancer. N Engl J Med.

[3] Huang SH, Xu W, Waldron J, Siu L, Shen X, Tong L (2015). Refining American Joint Committee on Cancer/Union for International Cancer Control TNM Stage and Prognostic Groups for Human Papillomavirus-related oropharyngeal carcinomas. J Clin Oncol.

[4] Rietbergen MM, Brakenhoff RH, Bloemena E, Witte BI, Snijders PJ, Heideman DA (2013). Human papillomavirus detection and comorbidity: critical issues in selection of patients with oropharyngeal cancer for treatment De-escalation trials. Ann Oncol.

[5] Machtay M, Moughan J, Trotti A, Garden AS, Weber RS, Cooper JS (2008). Factors associated with severe late toxicity after concurrent chemoradiation for locally advanced head and neck cancer: an RTOG analysis. J Clin Oncol.

[6] Wilson JA, Carding PN, Patterson JM (2011). Dysphagia after nonsurgical head and neck cancer treatment: patients’ perspectives. Otolaryngol Head Neck Surg.

[7] Patterson JM, Rapley T, Carding PN, Wilson JA, McColl E (2013). Head and neck cancer and dysphagia; caring for carers. Psychooncology.

[8] Haughey BH, Hinni ML, Salassa JR, Hayden RE, Grant DG, Rich JT (2011). Transoral laser microsurgery as primary treatment for advanced-stage oropharengeal cancer: a United States multicenter study. Head Neck.

[9] Moore EJ, Hinni ML (2013). Critical review: transoral laser microsurgery and robotic assisted surgery for oropharynx cancer including Human papillomavirus-related cancer. Int J Radiat Oncol Biol Phys.

[10] O’Hara J, Cosway B, Muirhead C, Leonard N, Goff D, Patterson J. Transoral laser microsurgery ± adjuvant therapy versus chemoradiotherapy for stage III and IVA oropharyngeal squamous cell carcinoma: Preliminary comparison of early swallowing outcomes. Head Neck. 2014; doi:10.1002/hed.23790. 10.1002/hed.23790 24891273

[11] Ang KK, Trotti A, Brown BW, Garden AS, Foote RL, Morrison WH (2001). Randomised trial addressing risk features and time factors of surgery plus radiotherapy in advanced head-and-neck cancer. Int J Radiat Oncol Biol Phys.

[12] Sinha P, Lewis JS, Piccirillo JF, Kallogieri D, Haughey BH (2012). Extracapsular spread and adjuvant therapy in Human papillomavirus-related, p16-positive oropharyngeal carcinoma. Cancer.

[13] Kramer S, Gelber RD, Snow JB, Marcial VA, Lowry LD, Davis LW (1987). Combined radiation therapy and surgery in the management of advanced head and neck cancer: final report of study 73–03 of the radiation therapy oncology group. Head Neck Surg.

[14] Peters LJ, Goepfert H, Ang KK, Byers RM, Maor MH, Guillamondequi O (1993). Evaluation of the dose for postoperative radiation therapy of head and neck cancer: first report of a prospective ramdomised trial. Int J Radiat Oncol Biol Phys.

[15] Kimple RJ, Smith MA, Blitzer GC, Torres AD, Martin JA, Yang RZ (2013). Enhanced radiation sensitivity in HPV-positive head and neck cancer. Cancer Res.

[16] Rieckmann T, Tribius S, Grob TJ, Meyer F, Busch CJ, Petersen C (2013). HNSCC cell lines positive for HPV and p16 possess higher cellular radiosensitivity due to an impaired DSB repair capacity. Radiother Oncol.

[17] Marur S, Lee JW, Cmelak A, Zhao W, Westra WH, Chung CH (2012). ECOG 1308: a phase II trial of induction chemotherapy followed by cetuximab with low dose versus standard dose IMRT in patients with HPV-associated resectable squamous cell carcinoma of the oropharynx (OP). J Clin Oncol.

[18] Bedi M, Firat S, Semenenko VA, Schultz C, Tripp P, Byhardt R (2012). Elective lymph node irradiation with intensity-modulated radiotherapy: is conventional dose fractionation necessary?. Int J Radiat Oncol Biol Phys.

[19] Cooper JS, Pajak TF, Forastiere AA, Jacobs J, Campbell BH, Saxman SB (2004). Postoperative concurrent radiotherapy and chemotherapy for high-risk squamous-cell carcinoma of the head and neck. N Engl J Med.

[20] Bernier J, Domenge C, Ozsahin M, Matuszewska K, Lefebvre JL, Greiner RH (2004). Postoperative irradiation with or without concomitant chemotherapy for locally advanced head and neck cancer. N Engl J Med.

[21] Bernier J, Cooper JS, Pajak TF, van Glabbeke M, Bourhis J, Forastiere A (2005). Defining risk levels in locally advanced head and neck cancers: a comparative analysis of concurrent postoperative radiation plus chemotherapy trials of the EORTC (#22931) and RTOG (#9501). Head Neck.

[22] Rackley T, Caley A, Palaniappan N, Evans M (2014). Management of oropharyngeal cancer – UK survey shows variations in practice. Clin Oncol.

[23] Eisbruch A, Kim HM, Feng FY, Lyden TH, Haxer MJ, Feng M (2011). Chemo-IMRT of oropharyngeal cancer aiming to reduce dysphagia: swallowing organs late complication probabilities and dosimetric correlates. Int J Radiat Oncol Biol Phys.

[24] Schwartz DL, Hutcheson K, Barringer D, Tucker SL, Kies M, Holsinger FC (2010). Candidate dosimetric predictors of long-term swallowing dysfunction after oropharyngeal intensity-modulated radiotherapy. Int J Radiat Oncol Biol Phys.

[25] Hutcheson KA, Holsinger FC, Kupferman ME, Lewin JS (2015). Functional outcomes after TORS for oropharyngeal cancer: a systematic review. Eur Arch Otorhinolaryngol.

[26] Steiner W, Ambrosch P (2000). Endoscopic laser surgery of the upper aerodigestive tract: with special emphasis on cancer surgery.

[27] The Royal College of Radiologists (2008). The timely delivery of radical radiotherapy: standards and guidelines for the management of unscheduled treatment interruptions.

[28] Gregoire V, Ang K, Budach W, Grau C, Hamoir M, Langendijk JA (2014). Delineation of the neck node levels for head and neck tumors: A 2013 update. DAHANCA, EORTC, HKNPCSG, NCIC CTG, NCRI, RTOG, TROG consensus guidelines. Radiother Oncol.

[29] Christianen ME, Langendijk JA, Westerlaan HE, van de Water TA, Bijl HP (2011). Delineation of organs at risk involved in swallowing for radiotherapy treatment planning. Radiother Oncol.

[30] Hutcheson KA, Lisec A, Denise A, Barringer MS, Portwood M, Lewin JS (2012). What is a clinically relevant difference in MDADI scores in head and neck cancer patients? Poster presentation at the American Head and Neck Society 8^th^ international conference on Head and Neck Cancer, Toronto, Canada.

[31] Dowthwaite SA, Franklin JH, Palma DA, Fung K, Yoo J, Nichols AC (2012). The role of transoral robotic surgery in the management of oropharyngeal cancer: a review of the literature. ISRN Oncol.

[32] Adelstein DJ, Ridge JA, Brizel DM, Holsinger FC, Haughey BH, O’Sullivan B (2012). Transoral resection of pharyngeal cancer: summary of a National Cancer Institute Head and Neck Cancer Steering Committee Clinical Trials Planning Meeting, November 6-7, 2011, Arlington, Virginia. Head Neck.

[33] Martin-Harris B, Brodsky MB, Michel Y, Castell DO, Schleicher M, Sandidge J (2008). MBS measurement tool for swallow impairment-MBSImp: establishing a standard. Dysphagia.

[34] Rosenbek JC, Robbins JA, Roecker EB, Coyle JL, Wood JL (1996). A penetration-aspiration scale. Dysphagia.

